# Platelet Count and Volume and Pharmacological Closure with Paracetamol of Ductus Arteriosus in Preterm Infants

**DOI:** 10.3390/children9010089

**Published:** 2022-01-10

**Authors:** Carlo Dani, Martina Ciarcià, Francesca Miselli, Michele Luzzati, Caterina Coviello, Angela Paladini, Anthea Bottoni, Vito D’Andrea, Giovanni Vento

**Affiliations:** 1 Department of Neurosciences, Psychology, Drug Research and Child Health, University of Florence, 50134 Florence, Italy; 2 Division of Neonatology, University of Florence, 50134 Florence, Italy; martina.ciarcia@unifi.it (M.C.); francesca.miselli@unifi.it (F.M.); michele.luzzati@unifi.it (M.L.); covielloc@aou-careggi.toscana.it (C.C.); 3 Division of Neonatology, Department of Woman and Child Health and Public Health, Fondazione Policlinico Universitario A. Gemelli, IRCCS, Catholic University of Sacred Heart, 20123 Rome, Italy; angela.paladini@unicatt.it (A.P.); anthea.bottoni@unicatt.it (A.B.); vito.dandrea@unicatt.it (V.D.); giovanni.vento@unicatt.it (G.V.)

**Keywords:** ductus arteriosus, paracetamol, platelets, preterm infants

## Abstract

**Background:** Low platelet count might promote resistance to pharmacological closure with indomethacin and ibuprofen of a hemodynamically significant patent ductus arteriosus (hsPDA). However, no studies have investigated if this occurs with paracetamol. **Methods:** We retrospectively assessed the correlation between platelet count, mean platelet volume (MPV), and plateletcrit (PCT), as well as the effectiveness of paracetamol in closing hsPDA in infants born at 23^+0^–31^+6^ weeks of gestation who were treated with 15 mg/kg/6 h of i.v. paracetamol for 3 days. **Results:** We studied 79 infants: 37 (47%) Had closure after a course of paracetamol and 42 (53%) did not. Platelet count and PCT did not correlate with paracetamol success or failure in closing hsPDA, while MPV was lower at birth (10.7 ± 1.4 vs. 9.5 ± 1.1; *p* < 0.001) and prior to starting therapy (11.7 ± 1.9 vs. 11.0 ± 1.6; *p* = 0.079) in refractory infants. Regression analysis confirmed that the low MVP measured prior to starting the treatment increased the risk of hsPDA paracetamol closure failure (OR 1.664, 95% CI 1.153–2.401). **Conclusions:** The greater MPV correlated positively with the effectiveness of paracetamol in closing hsPDA, while platelet count and PCT did not influence closure rates. Additional studies are needed to confirm our results.

## 1. Introduction

Patent ductus arteriosus (PDA) is a common disease of preterm infants who suffer from respiratory distress syndrome (RDS). Medical and/or surgical treatment for a hemodynamically significant PDA (hsPDA) is required for 60–70% of preterm infants below 28 weeks’ gestation [1]. There is still controversy concerning the proper management of PDA, since randomized controlled trials (RCTs) of hsPDA closure administering non-steroidal anti-inflammatory drugs (NSAIDs) have often failed to prove significant advantages in preterm infants [2]. Although infants who exhibit a persistent left-to-right shunt through the ductus arteriosus complicate RDS, they present an increased risk of respiratory failure, bronchopulmonary dysplasia (BPD), intraventricular hemorrhage (IVH), and mortality [1,3,4,5,6]. Indomethacin or ibuprofen is commonly used to perform the pharmacological closure. Both of the drugs are successful in promoting ductal closure in 70–80% of cases. However, severe adverse effects, such as gastrointestinal perforation, acute renal failure, and bleeding disorders have been reported [7,8,9,10]. Recent studies have demonstrated that paracetamol is effective in closing and constricting hsPDA [11,12,13] with a better safety profile [13,14,15].

Studies in animals have shown that low platelet count was associated with a higher risk of PDA in preterm infants, and with the failure of pharmacological closure, by limiting the thrombosis of ductal lumen during physiological anatomical closure [16]. On the other hand, a better luminal occlusion was promoted by a higher platelet count that indeed supports thrombosis [16]. Several clinical studies have recently confirmed these assumptions, demonstrating that low platelet count can augment the risk of developing hsPDA in preterm infants [17,18] and might promote its resistance to pharmacological closure with indomethacin and ibuprofen [19]. Moreover, other platelet indices, such as mean platelet volume (MPV) and plateletcrit (PCT), have been correlated with the development of hsPDA and its response to drug therapy [18,20].

However, no previous study has investigated the possible association between the effectiveness of paracetamol in closing hsPDA and platelet indices in very preterm infants. Therefore, the purpose of this study was to assess the possible correlation between platelet count, MPV, and PCT, as well as the effectiveness of paracetamol in promoting the closure of hsPDA in very preterm infants.

## 2. Materials and Methods

Herein, we performed this retrospective study at the third level neonatal intensive care unit (NICU) of Careggi University Hospital of Florence and Fondazione Policlinico Universitario A. Gemelli of Rome. Local ethics committees approved the study.

Infants with a gestational age ranging from 23^+0^ to 31^+6^ weeks, who developed a hsPDA which was treated with 15 mg/kg/6 h of i.v. paracetamol for 3 days [12,13], as the first pharmacological course or after the failure of an initial course of i.v. ibuprofen (Pedea^®^, Orphan Europe, Paris, France; 10 mg/kg followed by 5 mg/kg after 24 and 48 h) were enrolled. Exclusion criteria were major congenital malformations, chromosomal disorders, inherited metabolic diseases, death within the first week of life, and the lack of platelet data required for the study.

### 2.1. Platelet Measurements

We evaluated the platelet count, MPV, and PCT (=platelet count × MPV/10.000) at birth (within the first 3 h of life) and 24–48 h prior to starting the first course of paracetamol, since these measurements have the greatest probability of correlation with PDA response to paracetamol. A complete blood count was obtained from the samples by venous umbilical catheter, venipuncture or rarely arterial puncture.

### 2.2. PDA Diagnosis and Treatment

The diagnosis of hsPDA was made between 24–72 h of life by an echocardiographic presence of a ductal left-to-right shunt, with a left atrium-to-aortic root ratio >1.3 or a ductal size >1.5 mm and by eliminating the cases of restrictive PDA indicated using the closing flow pattern [13]. Infants were treated with paracetamol as a first choice when they had contraindications to ibuprofen (serum creatinine concentration >1.5 mg/dL, urine output <1 mL/kg/h or <0.5 mL/kg/h during the first 24 h of life; platelet count <50,000/mm^3^, IVH ≥ 3° grade), and as second choice when they were refractory to ibuprofen therapy.

Echocardiography was repeated as a minimum after 24 h from the last dose of the first pharmacological course and, subsequently, according to the PDA progress. A pediatric cardiologist or a neonatologist who has achieved adequate expertise in newborn heart ultrasound performed the cardiac ultrasounds.

When the PDA was still hemodynamically significant after the first pharmacological course, a second course of paracetamol or ibuprofen was administered 24 h after the last dose of the previous course. The successive pharmacological and/or surgical management of PDA was decided according to the local protocols.

The daily clinical care of the patients followed the common practice of each center. The fluid intake began at 70–80 mL/kg and was gradually augmented by 10–20 mL/kg/day, according to the changes in body weight, serum sodium concentrations, and osmolality, in order to achieve at the end of the first week of life the intake of 150–160 mL/kg. In the case of systemic hypotension which is resistant to fluid-replacement therapy, the dopamine and/or dobutamine treatment was administered. The treatment of RDS included oxygen therapy, respiratory support, and rescue surfactant replacement, in order to reach the following targets: PaO_2_ 50–60 mmHg, PaCO_2_ < 65 mmHg, pH > 7.20, SpO_2_ 90–95%.

### 2.3. Recording of Clinical Data

The medical and nursing chart review was carried out and for each infant the following data were recorded: Gestational age, birth weight, birth weight <10° percentile, sex, Apgar score at 5 min, mode of delivery, antenatal steroids, RDS occurrence, surfactant therapy, need and duration of non-invasive respiratory support (high-flow nasal cannulae (HFNC), nasal continuous airway pressure (NCPAP), bi-level NCPAP (BiPAP), nasal intermittent mechanical ventilation (N-IMV)) and mechanical ventilation (patient-triggered ventilation or high frequency ventilation (HFV)), treatment of vasoactive drugs (i.e., dopamine, dobutamine), occurrence of PDA requiring treatment with ibuprofen or surgical closure, sepsis, bronchopulmonary dysplasia (BPD), surgical necrotizing enterocolitis (NEC), IVH ≥ 3° grade, periventricular leukomalacia (PVL), retinopathy of prematurity (ROP) ≥ 3° grade, duration of hospital stay, and death. BPD was diagnosed in the case of oxygen need at 36 weeks post-menstrual age [21]. NEC was defined in agreement with Bell’s criteria [22]. In addition, the diagnosis of sepsis was made in the case of positive blood culture. IVH was graded following the classification of Papile et al. [23], and PVL was established in agreement with the De Vries’ criteria [24]. Furthermore, the severity of ROP was evaluated according to the International Classification [25].

### 2.4. Statistical Analysis

The infants enrolled in the study were stratified into subgroups of infants who had hsPDA closure after the first 3-day course of paracetamol and infants who did not. Their clinical characteristics were described as the mean and standard deviation, median and range or rate and percentage. The mean imputation was used to replace the missing data with an estimated value based on the other available information. Shapiro-Wilk’s test was applied to assess the normality of data distribution. Student’s “*t*” test was used to analyze the parametric continuous variables and the Wilcoxon rank sum test was used in the case of deviation from normality assumptions. The Χ^2^ test was used to compare categorical variables. *p* < 0.05 was considered statistically significant.

A multivariable stepwise logistic regression analysis was executed to assess the potential effect of independent variables on the effectiveness of paracetamol on hsPDA closure. Variables that upon univariate analyses were different between the groups (*p* < 0.100) were included in the model, excluding those who showed collinearity (variance inflation factors <5). Therefore, we studied the effects of gestational age, cesarean section, dopamine treatment, and MPV at birth and before the treatment with paracetamol. Results are presented as coefficients of independent variables with 95% confidence intervals (CI).

## 3. Results

The study was carried out from November 2017 to March 2020. We enrolled a total of 79 infants who developed hsPDA 37 (47%) who had closure after a course of paracetamol and 42 (53%) who did not. Paracetamol was given as first line of treatment in 55 (70%) patients or after failure of an initial course of ibuprofen in 24 (30%) patients at a mean age of 5.2 ± 6.8 days of life.

The clinical characteristics of infants responding and refractory to paracetamol are reported in Table 1: Infants refractory to paracetamol had lower gestational age, less frequent birth by caesarean section, and more frequent treatment with dopamine.

Upon univariate analysis, platelet count and PCT were similar in infants who responded or did not respond to paracetamol, both at birth and prior to starting therapy, while MPV was lower at birth (10.7 ± 1.4 vs. 9.5 ± 1.1 fL; *p* < 0.001) and prior to starting therapy (11.7 ± 1.9 vs. 11.0 ± 1.6 fL; *p* = 0.079) in refractory infants (Table 2).

Regression analysis demonstrated that MPV measured prior to starting the treatment with paracetamol increased the risk of hsPDA closure failure (OR 1.664, 95% Cl 1.153–2.401; *p* = 0.012), after adjusting for effects of gestational age, cesarean section, and dopamine treatment (Table 3).

## 4. Discussion

Our study is the first to investigate the possible correlation between platelet count, MPV, and PCT, as well as the pharmacological effect of paracetamol in closing hsPDA in very preterm infants. Our results demonstrated that platelet count and PCT, measured at birth and prior to starting therapy, did not affect paracetamol effectiveness, while smaller platelets prior to starting therapy increased the risk of paracetamol failure in closing hsPDA.

A recent meta-analysis on the pharmacological closure of hsPDA suggested that the pre-treatment with thrombocytopenia was associated with higher odds of failure of the first course of indomethacin or ibuprofen [19]. Our results disagreed with these findings. However, the meta-analysis did not include studies using paracetamol and thus could not be considered conclusive, since it reviewed eight heterogeneous studies (six retrospective studies and two case-control studies reporting conflicting results), which were mostly not primarily designed to address the issue of correlation between platelet count and pharmacological closure of hsPDA [19]. Moreover, a small percentage (11%) of our population had a moderate-to-severe thrombocytopenia (platelet count < 100 × 10^3^/mm^3^) and this might have made it difficult to demonstrate a possible correlation between thrombocytopenia and paracetamol failure. However, another intriguing possibility is that the effectiveness of paracetamol is actually less affected by platelet count in comparison with indomethacin and ibuprofen. In fact, both of these drugs are known to inhibit thromboxane release and impair platelet aggregation (enhancing the effect of thrombocytopenia in favoring the development of hsPDA), while paracetamol has been reported to have a negligible effect on platelet aggregation, making its closing effect on hsPDA less easily influenced by thrombocytopenia [26].

We found that the larger platelet volume prior to starting therapy decreased the risk of paracetamol failure in closing hsPDA. This might occur due to the fact that a larger MPV is known to enhance platelet reactivity promoting the activation of the coagulation system, and for this reason, facilitating the deposition of fibrin in the microcirculation [27]. Therefore, it is possible that larger platelets facilitate DA thrombosis and anatomical closure, enhancing the closing effect of paracetamol. Nevertheless, the effects of platelet volume have not been deeply explored in preterm infants: A few studies have compared the platelet volume of term and preterm infants with conflicting results, since MPV values were found to be comparable [28,29] or lower [30] in preterm than in term infants. Gioia et al. found that oxygen therapy or the need for mechanical ventilation at 48 h of life were more frequently associated with lower MPV [31], while other authors reported higher MPV in preterm infants with RDS [32] and BPD [20] compared to the control groups. However, the complex mechanisms which control the platelet size have not been completely elucidated. In addition, the inverse correlation between platelet count and volume, which has been described in preterm infants [32] (a low platelet volume might be associated with a high platelet count and vice versa) could reduce the potential effect of these platelet indices on hsPDA closure.

The limitations of the present study include the retrospective design and the relatively small size population, which precluded the possibility of evaluating other factors potentially influencing the effect of paracetamol in closing hsPDA, such as the treatment with other drugs (i.e., furosemide and aminoglycosides, which were found to have a vasodilatory effect in the DA of animal models) and genetics. However, considering the homogeneity of our population, it is improbable that our results are a statistical artefact resulting from a population substructure.

## 5. Conclusions

In this paper, we found that a greater platelet volume was positively correlated with the effectiveness of paracetamol in closing hsPDA, while platelet count and PCT were not. Additional studies on this topic are needed to confirm our results and to evaluate the clinical values of this biomarker in predicting the response of hsPDA to the pharmacological treatment with paracetamol.

## Figures and Tables

**Table 1:** Clinical characteristics of infants with hsPDA responding or refractory to the treatment with paracetamol. Mean ± SD or rate and (%) or median and (range).

	PDA Responding to Paracetamol (*n* = 37)	PDA Refractory to Paracetamol (*n* = 42)	*p*
**Gestational age (weeks)**	27.4 ± 2.4	26.2 ± 2.3	0.026
**Birth weight (g)** **<10° percentile**	911 ± 321 7 (19)	830 ± 287 5 (12)	0.240 0.136
**Male**	21 (57)	25 (60)	0.690
**Apgar score at 5th min**	7 (4–8)	7 (6–9)	0.842
**Antenatal steroids**	25 (68)	26 (62)	0.832
**Caesarean section**	30 (81)	25 (60)	0.037
**NIV** **Duration (h)**	31 (84) 25.6 ± 24.6	36 (86) 32.6 ± 26.2	0.812 0.227
**MV** **Duration (h)**	25 (68) 15 ± 18	34 (81) 15 ± 16	0.172 1.000
**Surfactant**	34 (92)	38 (90)	0.825
**Dopamine** **Dobutamine**	17 (46) 9 (24)	29 (69) 13 (31)	0.038 0.512
**Paracetamol as first choice for hsPDA treatment**	25 (68)	30 (71)	0.931
**Age at paracetamol treatment (d)**	4.8 ± 4.9	5.5 ± 8.1	0.649
**Surgical closure of hsPDA**	0	10 (24)	N/A
**Sepsis**	22 (59)	21 (50)	0.400
**BPD**	20 (54)	20 (48)	0.386
**NEC**	3 (8)	1 (2)	0.247
**IVH** **≥ 3° grade**	10 (27)	15 (36)	0.407
**PVL**	5 (14)	7 (17)	0.700
**ROP ≥ 3° grade**	7 (19)	4 (10)	0.229
**Mortality**	10 (26)	6 (14)	0.160
**Hospital stay (d)**	93 ± 61	89 ± 46	0.741

hsPDA: hemodynamically significant patent ductus areteriosus; NIV: Noninvasive ventilation; MV: Mechanical ventilation; PDA: Patent ductus arteriosus; BPD: Bronchopulmonary dysplasia; NEC: Necrotizing enterocolitis; IVH: Intraventricular hemorrhage; PVL: Periventricular hemorrhage; ROP: retinopathy of prematurity.

**Table 2:** Platelet count, mean platelet volume (MPV), and plateletcrit (PCT) in infants with hsPDA responding or refractory to the treatment with paracetamol. Mean ± SD or rate and (%).

	PDA Responding to Paracetamol (*n* = 37)	PDA Refractory to Paracetamol (*n* = 42)	*p*
**Platelet count (×10^3^/mm^3^) at birth before paracetamol**	184.8 ± 81.8 201.3 ± 137.7	199.4 ± 82.6 215.9 ± 110.6	0.433 0.603
**Platelet count at birth:**			
**<50 (×10^3^/mm^3^)**	2 (5)	1 (2)	0.436
**51–99 (×10^3^/mm^3^)**	4 (11)	2 (5)	0.311
**100–150 (×10^3^/mm^3^)**	7 (19)	11 (26)	0.442
**Platelet count before paracetamol:**			
**<50 (×10^3^/mm^3^)**	0	1 (2)	0.345
**51–99 (×10^3^/mm^3^)**	6 (16)	2 (5)	0.092
**100–150 (×10^3^/mm^3^)**	7 (19)	11 (26)	0.442
**MPV (fL):**			
**at birth**	10.7 ± 1.4	9.5 ± 1.1	<0.001
**before paracetamol**	11.7 ± 1.9	11.0 ± 1.6	0.079
**PCT (%):**			
**at birth**	0.21 ± 0.09	0.21 ± 0.08	1.000
**before paracetamol**	0.26 ± 0.19	0.24 ± 0.12	0.161

**Table 3:** Regression analysis correlating the effect of selected variables with the effectiveness of paracetamol on hsPDA closure.

	Odds Ratio and (95% CI)	*p*
Gestational age	1.241 (0.974–1.579)	0.080
Cesarean section	2.714 (0.800–9.204)	0.109
Dopamine	0.345 (0.113–1.053)	0.062
MPV at birth	1.462 (0.893–2.393)	0.131
MPV before treatment	1.664 (1.153–2.401)	0.012

## Data Availability

All of the relevant data are within the manuscript.

## References

[1] Hamrick S.E., Hansmann G. (2010). Patent ductus arteriosus of the preterm infant. Pediatrics.

[2] El-Khuffash A., Weisz D.E., McNamara P. (2016). Reflections of the changes in patent ductus arteriosus management during the last 10 years. Arch. Dis. Child. Fetal Neonatal Ed..

[3] Brooks J.M., Travadi J.N., Patole S.K., Doherty D.A., Simmer K. (2005). Is surgical ligation of patent ductus arteriosus necessary? The Western Australian experience of conservative management. Arch. Dis. Child. Fetal Neonatal Ed..

[4] Bussmann N., Smith A., Breatnach C.R., McCallion N., Cleary B., Franklin O., McNamara P.J., El-Khuffash A. (2021). Patent ductus arteriosus shunt elimination results in a reduction in adverse outcomes: A post hoc analysis of the PDA RCT cohort. J. Perinatol..

[5] Liebowitz M., Clyman R.I. (2017). Prophylactic Indomethacin Compared with Delayed Conservative Management of the Patent Ductus Arteriosus in Extremely Preterm Infants: Effects on Neonatal Outcomes. J. Pediatr..

[6] Schena F., Francescato G., Cappelleri A., Picciolli I., Mayer A., Mosca F., Fumagalli M. (2015). Association between hemody-namically significant patent ductus arteriosus and bronchopulmonary dysplasia. J. Pediatr..

[7] Evans P., O’Reilly D., Flyer J.N., Soll R., Mitra S. (2021). Indomethacin for symptomatic patent ductus arteriosus in preterm infants. Cochrane Database Syst. Rev..

[8] Ohlsson A., Walia R., Shah S.S. (2020). Ibuprofen for the treatment of patent ductus arteriosus in preterm or low birth weight (or both) infants. Cochrane Database Syst. Rev..

[9] Hammerman C., Bin-Nun A., Kaplan M. (2012). Managing the Patent Ductus Arteriosus in the Premature Neonate: A New Look at What We Thought We Knew. Semin. Perinatol..

[10] Thomas R.L., Parker G.C., Van Overmeire B., Aranda J.V. (2004). A meta-analysis of ibuprofen versus indomethacin for closure of patent ductus arteriosus. Eur. J. Nucl. Med. Mol. Imaging.

[11] Ohlsson A., Shah P.S. (2015). Paracetamol (acetaminophen) for patent ductus arteriosus in preterm or low-birth-weight infants. Cochrane Database Syst. Rev..

[12] Dani C., Mosca F., Cresi F., Lago P., Lista G., Laforgia N., Del Vecchio A., Corvaglia L., Paolillo P., Trevisanuto D. (2019). Patent ductus arteriosus in preterm infants born at 23-24 weeks’ gestation: Should we pay more attention?. Early Hum. Dev..

[13] Dani C., Lista G., Bianchi S., Mosca F., Schena F., Ramenghi L., Zecca E., Vento G., Poggi C., Leonardi V. (2020). Intravenous paracetamol in comparison with ibuprofen for the treatment of patent ductus arteriosus in preterm infants: A randomized controlled trial. Eur. J. Pediatr..

[14] Marconi E., Bettiol A., Ambrosio G., Perduca V., Vannacci A., Troiani S., Dani C., Mugelli A., Lucenteforte E. (2019). Efficacy and safety of pharmacological treatments for patent ductus arteriosus closure: A systematic review and network meta-analysis of clinical trials and observational studies. Pharmacol. Res..

[15] Dani C., Poggi C., Cianchi I., Corsini I., Vangi V., Pratesi S. (2018). Effect on cerebral oxygenation of paracetamol for patent ductus arteriosus in preterm infants. Eur. J. Nucl. Med. Mol. Imaging.

[16] Echtler K., Stark K., Lorenz M., Kerstan S., Walch A., Jennen L., Rudelius M., Seidl S., Kremmer E., Emambokus N.R. (2010). Platelets contribute to postnatal occlusion of the ductus arteriosus. Nat. Med..

[17] Poggi C., Fontanelli G., Dani C. (2012). Relationship between Platelet Count and Volume and Spontaneous and Pharmacological Closure of Ductus Arteriosus in Preterm Infants. Am. J. Perinatol..

[18] Ding R., Zhang Q., Duan Y., Wang D., Sun Q., Shan R. (2021). The relationship between platelet indices and patent ductus arte-riosus in preterm infants: A systematic review and meta-analysis. Eur. J. Pediatr..

[19] Mitra S., Chan A.K., Paes B.A., on behalf of the Thrombosis and Hemostasis in Newborns (THIN) Group (2016). The association of platelets with failed patent ductus arteriosus closure after a primary course of indomethacin or ibuprofen: A systematic review and meta-analysis. J. Matern. Neonatal Med..

[20] Dani C., Poggi C., Barp J., Berti E., Fontanelli G. (2011). Mean platelet volume and risk of bronchopulmonary dysplasia and in-traventricular hemorrhage in extremely preterm infants. Am. J. Perinatol..

[21] Ehrenkranz R.A., Walsh M.C., Vohr B.R., Jobe A.H., Wright L.L., Fanaroff A.A., Wrage L.A., Poole K., for the National Institutes of Child Health and Human Development Neonatal Research Network (2005). Validation of the National Institutes of Health Consensus Definition of Bronchopulmonary Dysplasia. Pediatrics.

[22] Bell M.J., Ternberg J.L., Feigin R.D., Keating J.P., Marshall R., Barton L., Brotherton T. (1978). Neonatal necrotizing enterocolitis: Therapeutic decisions based on clinical staging. Ann. Surg..

[23] Papile L.A., Burstein J., Burstein R., Koffler H. (1978). Incidence and evolution of the subependymal intraventricular hemorrhage: A study of infants weighing less than 1500 grams. J. Pediatr..

[24] De Vries L.S., Eken P., Dubowitz L.M. (1992). The spectrum of leukomalacia using cranial ultrasound. Behav. Brain Res..

[25] Dani C., Lori I., Favelli F., Frosini S., Messner H., Wanker P., De Marini S., Oretti C., Boldrini A., Massimiliano C. (2011). Lutein and zeaxanthin supplementation in preterm infants to prevent retinopathy of prematurity: A randomized controlled study. J. Matern. Neonatal Med..

[26] Driver B., Marks D.C., Van Der Wal D.E. (2019). Not all (N)SAID and done: Effects of nonsteroidal anti-inflammatory drugs and paracetamol intake on platelets. Res. Pr. Thromb. Haemost..

[27] Yurdakök M., Yigit S. (1999). Hemostatic system in early respiratory distress syndrome: Reduced fibrinolytic state?. Turk. J. Pediatr..

[28] Arad I.D., Alpan G., Sznajderman S.D., Eldor A. (1986). The Mean Platelet Volume (MPV) in the Neonatal Period. Am. J. Perinatol..

[29] Wasiluk A., Osada J., Dabrowska M., Szczepański M., Jasinska E. (2009). Does prematurity affect platelet indices?. Adv. Med. Sci..

[30] Patrick C.H., Lazarchick J., Stubbs T., Pittard W.B. (1987). Mean Platelet Volume and Platelet Distribution Width in the Neonate. J. Pediatr. Hematol..

[31] Gioia S., Piazze J., Anceschi M.M., Cerekja A., Alberini A., Giancotti A., Larciprete G., Cosmi E.V. (2007). Mean platelet volume: Association with adverse neonatal outcome. Platelets.

[32] Canpolat F.E., Yurdakök M., Armangil D., Yiğit Ş. (2009). Mean platelet volume in neonatal respiratory distress syndrome. Pediatr. Int..

